# The Impact of the Low Molecular Weight Heparin Tinzaparin on the Sensitization of Cisplatin-Resistant Ovarian Cancers—Preclinical In Vivo Evaluation in Xenograft Tumor Models

**DOI:** 10.3390/molecules22050728

**Published:** 2017-05-03

**Authors:** Thomas Mueller, Daniel Bastian Pfankuchen, Kathleen Wantoch von Rekowski, Martin Schlesinger, Franziska Reipsch, Gerd Bendas

**Affiliations:** 1 Department of Internal Medicine IV, Oncology/Hematology, Martin-Luther-University Halle-Wittenberg, Ernst-Grube-Straße 40, 06120 Halle (Saale), Germany; thomas.mueller@medizin.uni-halle.de (T.M.); freipsch@gmail.com (F.R.); 2 Department of Pharmacy, University Bonn, An der Immenburg 4, 53121 Bonn, Germany; danielpfankuchen@uni-bonn.de (D.B.P.); Kathleen.Wantoch@uni-bonn.de (K.W.v.R.); martin.schlesinger@uni-bonn.de (M.S.)

**Keywords:** cisplatin, low molecular weight heparin (LMWH), ovarian cancer, resistance

## Abstract

Resistance formation of tumors against chemotherapeutics is the major obstacle in clinical cancer therapy. Although low molecular weight heparin (LMWH) is an important component in oncology referring to guideline-based antithrombotic prophylaxis of tumor patients, a potential interference of LMWH with chemoresistance is unknown. We have recently shown that LMWH reverses the cisplatin resistance of A2780cis human ovarian cancer cells in vitro. Here we address the question whether this LMWH effect is also valid under in vivo conditions. Therefore, we established tumor xenografts of A2780 and cisplatin resistant A2780cis cells in nude mice and investigated the impact of daily tinzaparin applications (10 mg/kg BW) on anti-tumor activity of cisplatin (6 mg/kg BW, weekly) considering the tumor growth kinetics. Intratumoral platinum accumulation was detected by GF-AAS. Xenografts of A2780 and A2780cis cells strongly differed in cisplatin sensitivity. As an overall consideration, tinzaparin co-treatment affected the response to cisplatin of A2780cis, but not A2780 tumors in the later experimental time range. A subgroup analysis confirmed that initially smaller A2780cis tumors benefit from tinzaparin, but also small A2780 xenografts. Tinzaparin did not affect cisplatin accumulation in A2780cis xenografts, but strongly increased the platinum content in A2780, obviously related to morphological differences in both xenografts. Although we cannot directly confirm a return of A2780cis cisplatin resistance by tinzaparin, as shown in vitro, the present findings give reason to discuss heparin effects on cytostatic drug efficiency for small tumors and warrants further investigation.

## 1. Introduction

Cancer-associated thrombosis is an important mortality factor in malignant tumor diseases [1]. Therefore, antithrombotic prophylaxis or treatment is a common component in the therapeutic regimes of patients with malignancies. Based on the current clinical guidelines for antithrombotic treatment of cancer patients, low molecular weight heparin (LMWH) is the drug of choice [2,3]. Consequently, tremendous amounts of clinical data exist concerning the clinical treatment of cancer patients with LMWH. The retrospective analysis of those clinical data implies that anticoagulation by LMWH could possess a survival benefit for certain cancer patients, which probably goes beyond solely an antithrombotic efficiency [4]. This postulate has been addressed by a high number of preclinical investigations during the last two decades to figure out the potential mode of action. LMWH was shown to affect the metastatic capacities of various carcinomas by interfering with different functional axes, such as cell adhesion [5], migration [6], chemokine and growth factor signaling [7] or the enzymatic activity of heparanase [8].

However, little is known whether and how LMWH affects the classical chemotherapeutic treatment of cancer. One can presume that attenuation of thrombosis formation close to the solid tumors increases the accessibility for chemotherapeutics and decreases the interstitial pressure, both leading to higher drug efficiency. A number of studies refer to those mechanisms and display a therapeutic effect for the combination of LMWH and chemotherapy [9,10,11]. A retrospective consideration of the combination of LMWH with chemotherapy in patients with small cell lung cancer suggest a protective effect of heparin [12].

Nevertheless, acquired tumor cell resistance against single or series of chemotherapeutics is the major obstacle in clinical treatment of cancer, such as ovarian carcinomas [13]. The molecular mechanisms of those chemoresistance phenomena are manifold and often less understood. Cisplatin, the standard drug for clinical treatment of ovarian cancer and several other tumor entities is also often restricted in its efficiency by resistance formation of tumors [14]. Consequently, chemoresistance appears as attractive target to overcome restrictions in chemotherapeutic treatment. However, findings for a potential interference of heparin application and chemoresistance are hardly known [15].

We have recently shown that cisplatin-resistant A2780cis ovarian cancer cells were “resensitized” for cisplatin cytotoxicity to the level of non-resistant A2780 cells by a therapeutic LMWH (tinzaparin) concentration in a preclinical in vitro setting [16]. Considering the outstanding role of LMWH in cancer treatment, this surprising and novel finding linking LMWH application in cancer treatment with a potential circumvention of chemoresistance could have a high clinical relevance. However, the molecular mechanisms are not fully elucidated. It was shown that tinzaparin has no effect on the intracellular drug levels thus the higher efficiency in the resistant cells is not simply related to transport mechanisms. A whole genome analysis of tinzaparin pretreated resistant A2780cis cells confirmed that tinzaparin possesses a complex impact on signaling and gene regulatory mechanisms reversing the resistance phenomenon. Obviously, this LMWH efficiency is related to the cellular proteoglycans.

In a further step to evaluate the value of this finding here we address the question whether the chemoresistance overcoming capacity of tinzaparin is potentially valid and detectable under in vivo conditions. Considering the strong differences between in vitro 2D-cytotoxicity assays and in vivo tumor growth models we aimed to draw a parallel by establishing A2780 and A2780cis tumor xenografts in mice and investigated their cisplatin treatment in response to tinzaparin.

## 2. Results

To investigate the efficiency of cisplatin treatment and the impact of tinzaparin co-treatment for tumor growth under in vivo conditions, xenograft tumors in nude mice were established using the A2780 and cisplatin-resistant A2780cis cell line. In both trials, tumor bearing mice were divided into four groups with a similar mean tumor volume (*n* = 8) at start of treatment. Furthermore, the groups were set up to contain small, middle and larger tumors at the start of treatment with similar distribution between the groups. Mice were treated with saline, tinzaparin, cisplatin or a combination of tinzaparin with cisplatin, respectively.

### 2.1. Comparison of the Xenograft Tumor Models

**Figure 1:**
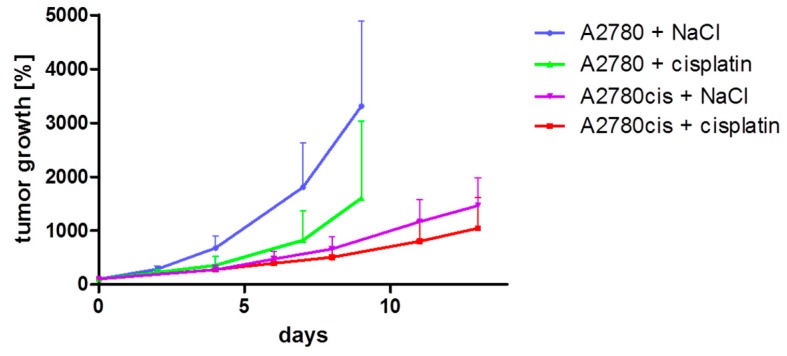
Comparison of A2780 and A2780cis tumor models regarding growth rate and response to cisplatin. Shown is the increase of mean tumor volumes of each group (*n* = 8) ± standard deviation normalized to day 0 (start of treatment). In the A2780 model, the treatment and monitoring of the saline control group was discontinued on day 9 after four mice had reached the maximal acceptable tumor volume. Mice of cisplatin group were monitored for a prolonged time, which is not depicted here. In the A2780cis model, monitoring of the control group was discontinued on day 15 when six mice had reached the maximal acceptable tumor volume whereas the cisplatin group was monitored for a prolonged time. The data depicted here are derived from the complete data sets shown in Figure 3 and Figure 4.

First, for a direct comparison of both tumor models regarding growth rate and response to cisplatin, the respective data of the saline- and cisplatin-groups were combined in one graph, which is shown in Figure 1. Overall, the A2780 tumors showed a higher growth rate compared to A2780cis tumors. A2780 tumors of the saline treated control group reached the maximal acceptable tumor volume much earlier than A2780cis tumors. Cisplatin treatment led to a clear growth inhibition effect compared to saline control in the A2780 model with a reduction of tumor growth of more than 50% when analyzed on days 7 and 9 (Figure 1). A much lower impact of cisplatin on tumor growth was observed in the A2780cis model with a growth inhibition of less than 30% (Figure 1). Thus, the in vitro cisplatin resistance of A2780cis cells was reproduced in vivo as xenograft tumors, although the relative difference in cisplatin sensitivity between both models turned out to be of lesser extent as compared to in vitro conditions.

**Figure 2:**
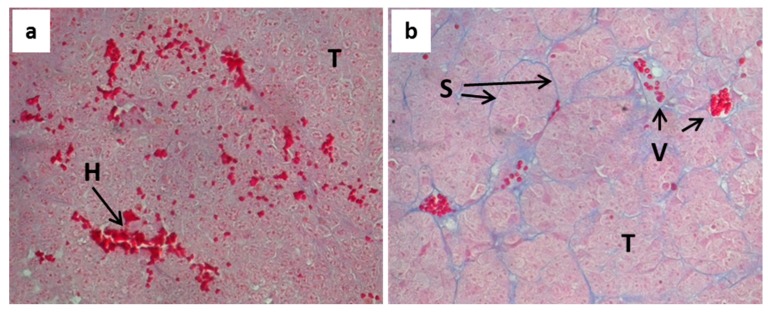
Histological analysis of tumor tissue of A2780 (**a**) and A2780cis tumors (**b**) after Azan-staining. A2780cis tumors show a characteristic composition of tumor cells together with septal structures. Particular high occurrence of hemorrhages is a typical feature of A2780 tumors. (T—tumor tissue, H—hemorrhage, S—septum, V—vessel; 400 × magnification).

To investigate whether the differences in growth rate of both tumor models were reflected by morphological differences, we investigated the structural peculiarities of both tumor models. Histological examination after HE staining revealed differences predominantly with regard to the vascularization. A2780cis tumors are highly vascularized and contain well-structured vessels of different shapes and diameters. A2780 tumors are even more vascularized, show structured vessels, but are also characterized by particular high occurrence of hemorrhages. This correlates with the red-blue colored appearance of A2780 tumors when subcutaneously grown in nude mice. For further characterization and comparison of both tumor models, we performed Azan-staining, which is useful to visualize extracellular matrix material and fibrous structures. This revealed an additional difference between both models. As shown in Figure 2, a typical feature of the A2780cis tumor tissue is a characteristic composition of tumor cells together with septal structures. Such tissue pattern is scarcely found in A2780 tumors.

### 2.2. Impact of Tinzaparin on Tumor Growth and Response to Cisplatin Treatment

**Figure 3:**
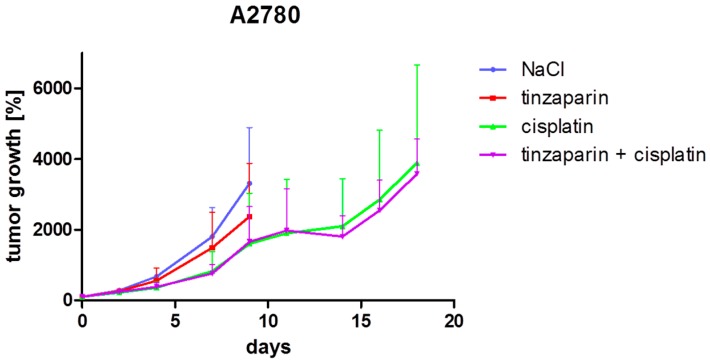
Analysis of tumor growth inhibition in the A2780 model. The increase of mean tumor volumes of each group (*n* = 8) ± standard deviation is illustrated normalized to day 0 (start of treatment). Tinzaparin was administered as daily injections of 10 mg/kg body weight. Cisplatin (6 mg/kg body weight) was given once weekly for two weeks. A third cisplatin injection was omitted due to incomplete recovery of mouse body weight after the second injection. Control group received saline. The treatment and monitoring of both the control group and the tinzaparin group was discontinued on day 9 after four mice had reached the maximal acceptable tumor volume, respectively. On day 11, two tumors in the cisplatin group and two tumors in the cisplatin/tinzaparin group had reached the maximal acceptable tumor volume and the mice were removed from the study. On day 14, further two mice of each group were removed. The remaining four mice of both groups were monitored until day 18.

**Figure 4:**
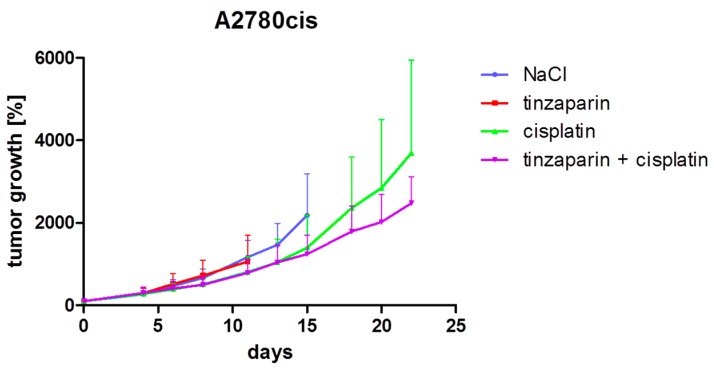
Analysis of tumor growth inhibition in the A2780cis model. Shown is the increase of mean tumor volumes of each group (*n* = 8) ± standard deviation normalized to day 0 (start of treatment). Tinzaparin was administered as daily injections of 10 mg/kg body weight. Cisplatin (6 mg/kg) was given once weekly for three weeks. Control group received saline. The treatment and monitoring of the tinzaparin group was discontinued on day 11 after four mice had reached the maximal acceptable tumor volume. Monitoring of control group was continued until day 15 when six mice had reached the maximal acceptable tumor volume. In addition, five tumors in the cisplatin group and five tumors in the cisplatin/tinzaparin group had reached the maximal acceptable tumor volume and the mice were removed from the study on day 15. The remaining three mice of both groups were monitored until day 22.

Tinzaparin was given as daily injections of 10 mg/kg body weight which was well tolerated; no weight loss or bleeding complications were observed. However, considering the combined graphs, treatment with tinzaparin alone resulted in a tumor growth inhibition effect in the A2780 model (Figure 3). In contrast, no tumor growth inhibition by tinzaparin alone was observed in the A2780cis model (Figure 4).

This obvious difference in tinzaparin effects in the two tumor models can be explained with a more detailed view on the individual mice data. Analyses of individual mice revealed a more heterogeneous tumor growth under tinzaparin treatment compared to treatment with saline, preferentially in the A2780 model. A2780 tumors with small initial volume seemed to be inhibited which explains the shift of the mean graph of the tinzaparin group (Figure 3). Interestingly, one of the tumors with smaller initial volume within the tinzaparin groups of both models did not grow or showed regression with no further growth. This was exclusively observed for the solely tinzaparin treated groups.

The combination of cisplatin as a once weekly application of 6 mg/kg body weight and tinzaparin did not result in an additional growth inhibition effect in both models, at least in an observation frame up to day 15. At this time point, part of the mice of each group had reached the maximal acceptable tumor volume and had to be removed from the study. Mice, which had not reached the maximal acceptable tumor volume at this time point, were maintained for the experiment. Therefore, a smaller number of mice (4 vs. 4 in A2780 and 3 vs. 3 in A2780cis) were monitored for an additional time period. This subgroup analysis revealed a difference between the two tumor models. An improved tumor growth inhibition effect of the combination treatment with cisplatin and tinzaparin became evident in the A2780cis model (Figure 4) but not in the A2780 tumor model (Figure 3).

**Figure 5:**
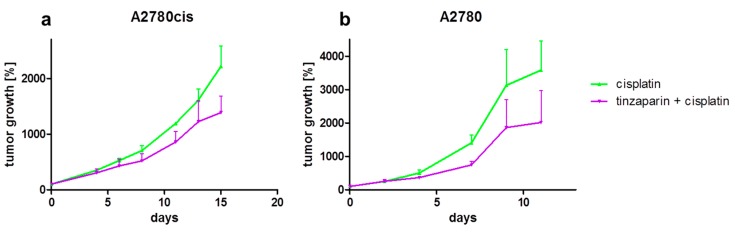
Impact of tinzaparin on cisplatin treatment of tumors with small initial volume. Shown is the increase of mean tumor volumes of each group (*n* = 3) ± standard deviation normalized to day 0 (start of treatment), (**a**) A2780cis and (**b**) A2780 xenografts. Tinzaparin and cisplatin treatment was as mentioned before.

The tumor growth inhibition in the later stages of experiment (Figure 4) refers to a preferential effect of the combined tinzaparin and cisplatin treatment on initially smaller tumors. To strengthen this postulation, a comparison of combination treatment vs. cisplatin alone was performed by separately analyzing the three tumors with the smallest initial volume from each group, respectively. This revealed a more evident effect of tinzaparin co-treatment on cisplatin induced growth inhibition in A2780cis model than in Figure 4 (Figure 5a), but A2780 xenografts (Figure 5b) also appear to benefit from tinzaparin when considering the smallest tumors, which was not expected from Figure 3.

### 2.3. The Impact of Tinzaparin On Tumor Platination

**Figure 6:**
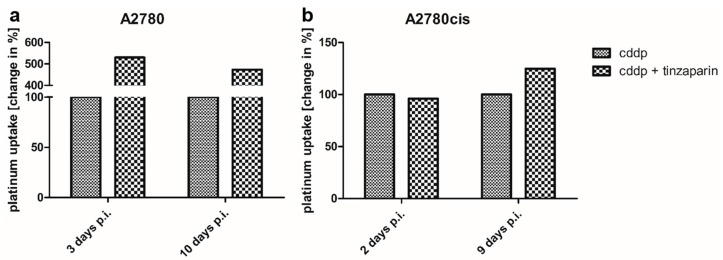
Detection of intratumoral platinum accumulation in A2780 (**a**) and A2780cis (**b**) tumor xenografts. Tumor samples were taken after 3 and 10 (A2780) or after 2 and 9 days (A2780cis) following the last cisplatin injection. The platinum concentration of tinzaparin treated tumors was normalized to only cisplatin treated tumors which were set to 100%, heparin induced changes were displayed in %.

In order to elucidate whether the tinzaparin treatment of mice had an impact on the cisplatin accumulation in the tumor tissue and thus higher cisplatin levels could explain the attenuated tumor growth kinetics in the A2780cis xenografts at the later time points, platinum concentrations were detected in the tumor tissue by the GF-AAS technique. Therefore, A2780 and A2780cis tumors were taken from tinzaparin-treated and -untreated mice at two different time points after the last cisplatin application. For each time point, the non-LMWH treated platinum concentration was set as 100% to individually illustrate changes in the platinum levels induced by heparin. It becomes evident that in A2780 tumors both, at day 3 and 10 post cisplatin injection the daily tinzaparin dosis affects drastically the platinum content (Figure 6a) showing four- to fivefold higher platinum concentrations. This is in contrast to our in vitro findings that displayed no effect of tinzaparin on cisplatin uptake by A2780 cells [16]. However, we cannot directly compare these findings since we have no information on the intratumor localization of the drug. Nevertheless, the higher platinum concentrations induced by tinzaparin is obviously not reflected by a higher overall cytotoxicity, as indicated in Figure 3.

In strict contrast, tinzaparin treatment had no or only a negligible impact on platinum accumulation in the cisplatin resistant tumor xenografts (Figure 6b). This is a clear indication that the slower tumor growth kinetics in the combined cisplatin and tinzaparin treatment compared to the cisplatin group at the later experimental time points is not related to higher drug concentrations within the tumor tissue.

One can assume that the strong differences in platinum accumulation reflect the morphological differences between both tumor xenografts, shown in Figure 2. In the A2780 model, which is characterized by a lesser organized tissue structure as compared to the A2780cis model, tinzaparin treatment could have impacted the intratumoral cisplatin distribution thereby resulting in improved accumulation.

## 3. Discussion

Considerable progress in elucidating beneficial effects of heparin treatment of cancer has been documented during the last two decades, which goes beyond the guideline-based application of LMWH to circumvent or treat venous thrombotic events [17,18]. Most attention has been given to the meanwhile experimentally confirmed fact that heparin impacts the metastatic spread of carcinomas [5]. In doing this, heparin possesses multiple “pleiotropic” effects to interfere with the tumor cell communication and the support, given by host or stroma components [19,20]. Consequently, heparin blocks tumors at an early development stage. However, little is known whether heparin can additionally affect tumors in an early stage of development or existing solid tumors, i.e., by assisting cytostatics. The cytostatic treatment of cancer patients is often hampered by the rapid development of cancer cell resistance against single or whole classes of cytostatic agents [21]. Despite heparin is a common constituent in clinical cancer therapy, a certain link between chemoresistance and heparin does not exist.

We could recently show in an in vitro approach that the LMWH tinzaparin reverses the cisplatin resistance in human A2780 ovarian cancer cells by a genetic reprogramming and a deregulation of more than 3700 genes, although cytotoxic or apoptotic effects of the LMWH doses used could be excluded [16]. The molecular mechanisms are still not elucidated and a matter of ongoing research.

Here we performed a first adaptation of this finding to in vivo relevant conditions and compare for the first time the cytostatic activity in a wild-type and cisplatin resistant subtype of a tumor cell line in relation to a LMWH therapeutic application. We provide evidence that the cisplatin resistance of A2780cis cells is maintained in the mice xenograft model, when compared to A2780 cell xenografts. However, the resistant cell xenografts display an overall slower growth kinetic than the wild-type ones and both tumor models show morphological differences, which requires a selective interpretation of cisplatin cytotoxicity response. This emphasizes impressively that the in vitro conditions of a plain 2D cultivation of pure tumor cell cultures are severely limited when transferred to experimental in vivo conditions, where the tumor cells are embedded and impacted by connective tissues, stroma cells. Furthermore, their access to the drugs is thus not a simple diffusion process but mediated and restricted by the blood vasculature.

Based on these differences in growth rate and morphology between the xenografts of A2780 and A2780cis we could not simply expect a sensitization of A2780cis cells for cisplatin by tinzaparin resulting in a similar behavior like the A2780 cells, as the cells adapted in vitro. Nevertheless, the overall consideration of tumor growth kinetics referred to a slightly diminished growth rate for A2780cis after day 15 in the tinzaparin and cisplatin combination group (Figure 4). This is a clear, but not a significant indication for improved cytotoxic activity in presence of tinzaparin. It turns out that the initially smallest A2780cis tumors are mostly influenced by the tinzaparin application and display the most evident growth retardation. Since tinzaparin hardly affected the cisplatin accumulation in the A2780cis xenografts (Figure 6) and did not display an intrinsic effect on growth inhibition (Figure 4) this could point to a higher sensitivity for cisplatin as reason for growth reduction.

The smallest A2780 tumors also displayed a slower growth rate co-treated with cisplatin and tinzaparin when compared to the solely cisplatin treated groups. However, the background of this type of “sensitization” seems different from A2780cis, since A2780 xenografts accumulated massively higher amounts of cisplatin in the tumor tissue by tinzaparin activity, obviously enforced by the leaky tissue structure. We can presently not explain completely the reason for this difference in cisplatin vascular leakage triggered by tinzaparin between A2780 and A2780cis. Since both xenografts are highly vascularized, the much higher leakage in A2780 xenografts seems to be related to the hemorrhagic regions and thus an even higher intratumoral bleeding when heparinized, and not a functional effect of tinzaparin on intact vasculature. However, the up to fivefold higher cisplatin concentrations in A2780 tumor tissue in presence of tinzaparin appear as the primary reason for slower growth, although intrinsic effects of tinzaparin alone might also play a role, as indicated in Figure 3.

Despite these obvious differences in A2780 and A2780cis concerning morphology and uptake characteristics and the different background of the term “sensitization” in both models, it is interesting to point out that only the tumors in early stages are susceptible for LMWH effects, as indicated for the anti-metastatic approaches with heparin.

Our postulations on a sensitization of tumors for chemotherapy do hardly find clear reflections by existing clinical data, since the classical data for clinical outcomes like progression free survival or overall survival probably also cover a potential better response to cytostatics. However, our postulations might be in line with those studies which explicitly focus on cytostatic efficiency [9,10,12] that proposed a beneficial effect of LMWH. These findings warrant further investigations to probably open new aspects for heparin applications in oncology.

## 4. Materials and Methods

### 4.1. Cell Lines

A2780 and cisplatin-resistant A2780cis human ovarian carcinoma cell lines were from the ECACC (Salisbury, UK); No. 93112519 (A2780) and No. 93112517 (A2780cis) and cultivated (37 °C, 5% CO_2_) in RPMI1640 medium containing 10% FCS, 1.5% L-glutamine and 1% penicillin/streptomycin (PAN Biotech, Aidenbach, Germany). After purchase, cells were frozen in aliquots (master cell bank) from which they were cultivated for a maximum of ten passages for the present study. Cell authentication was confirmed by short tandem repeat (STR) profiling.

### 4.2. Animal Studies

The investigations of this study were approved by the Laboratory Animal Care Committee of Sachsen-Anhalt, Germany. Xenograft tumors were generated in athymic nude mice (Charles River, Sulzfeld, Germany) using the ovarian carcinoma cell lines A2780 and A2780cis. Eight million cells of either cell line were resuspended in PBS and injected subcutaneously into the flank of mice. After establishment of tumors, mice were divided into four groups with a similar mean tumor volume (*n* = 8) at start of treatment and with similar distribution of tumor volumes between the groups. Tinzaparin was given as daily i.p. injections of 10 mg/kg body weight. Cisplatin (6 mg/kg BW) was administered i.p. once weekly. The control group received normal saline. Tumor growth and response to therapy was monitored by caliper measurements and tumor volume calculation using the formula a^2^ × b × 0.5 with a being the short and b the long dimension.

### 4.3. Tumor Lysis and Measurement of Intratumoral Platinum Accumulation

The kinetic of cisplatin uptake by A2780 and A2780cis tumors was analyzed by graphite furnace atomic absorption spectrometry (GF-AAS). Tumor samples were taken at the indicated time points and snap frozen. At time of detection, tumors were dissected with a scalpel, lysed with 65% nitric acid suprapur^®^ (Merck Chemicals, Schwalbach, Germany) for one hour at 80 °C followed by GF-AAS investigation, as indicated before [16]. Measured platinum concentrations were related to the tumor weights.

### 4.4. Histological Analyses

For hematoxylin and eosin (HE) staining, necropsied tumors were cross-sectioned, fixed in 4% formalin, embedded in paraffin, sliced with a RM 2245 microtome (4–5 μm, Leica, Wetzlar, Germany), dewaxed and rehydrated by decreasing alcohol series from xylene up to bi-distilled water. The slices were stained with hematoxylin (Dako, Hamburg, Germany), followed by several washing steps with tap water and bi-distilled water. Subsequently, the slices were stained with eosin (Merck Chemicals GmbH, Darmstadt, Germany). After staining, the slices were dehydrated by ascending alcohol series and fixed with Roti^®^-Histokit (Carl Roth GmbH & Co. KG, Karlsruhe, Germany).

For Azan staining, the dewaxed and rehydrated tissue slices were initially stained with an azocarmine (Morphisto^®^, Frankfurt a.M., Germany) solution. After some washing steps with bi-distilled water and the nuclei differentiation with aniline (Baacklab^®^, Schwerin, Germany) in 95% ethanol, the slices were treated with 5% phosphomolybdic acid (Baacklab^®^). Subsequently they were rinsed with bi-distilled water and stained with a solution of aniline blue and Orange G (Baacklab^®^). Afterwards the slices were washed with bi-distilled water, dehydrated by ascending alcohol series and fixed with Roti^®^-Histokitt (Carl Roth GmbH & Co. KG).
